# The Effect of Early Goal-Directed Therapy On Outcome in Adult Severe Sepsis And Septic Shock Patients: A Meta-Analysis of Randomised Clinical Trials

**DOI:** 10.1186/2197-425X-3-S1-A47

**Published:** 2015-10-01

**Authors:** J Xu, Y Yang, H Qiu

**Affiliations:** Southeast University, Zhongda Hospital, Nanjing, China

## Background

The controversial question of whether early goal-directed therapy (EGDT) improves outcome of severe sepsis and septic shock is very relevant and topical. Our goal was to examine whether EGDT improved outcome when employed in the resuscitation of adult sepsis patients compared with control care by meta-analysis.

## Methods

We searched for and gathered data from MEDLINE, Elsevier, Cochrane Central Register of Controlled Trials and Web of Science databases. Studies were eligible if they compared the effects of EGDT versus control care on mortality in adult patients with severe sepsis and septic shock. Two reviewers extracted data independently. Data including mortality, sample size of the patients with severe sepsis and septic shock, and resuscitation endpoints were extracted. Data were analyzed by the methods recommended by the Cochrane Collaboration Review Manager 4.2 software.

## Results

Eight studies compared EGDT with control care, and 3959 severe sepsis and septic shock patients were included. a trend towards reduced all-cause mortality was observed in EGDT group compared with control care (RR 0.86; 95% CI, 0.73 to 1.02; *p* = 0.08). For patients with a higher severity of disease (acute physiology and chronic health evaluation II score ≥ 20), EGDT reduced mortality significantly (RR 0.87; 95% CI, 0.77 to 0.99; p = 0.03) when compared with control care. Moreover, EGDT significantly reduced ICU mortality in severe sepsis and septic shock patients (RR 0.72; 95% CI, 0.61 to 0.85; *p* = 0.0002). Predefined subgroup analysis according to protocol with versus without ScvO_2_ suggested that mortality benefit appears with ScvO_2_ monitoring (RR 0.78; 95% CI, 0.66 to 0.91; *p* = 0.002) when compared with protocol including identical remaining intervention goals.

## Conclusions

EGDT reduced mortality in septic shock patients with a higher severity of disease when comparing to control care. Furthermore, when compared with resuscitation protocols without ScvO_2_ monitoring, EGDT goals called for CVP, MAP and ScvO_2_ showed a significant mortality benefit.

